# PRAS40 and PRR5-Like Protein Are New mTOR Interactors that Regulate Apoptosis

**DOI:** 10.1371/journal.pone.0001217

**Published:** 2007-11-21

**Authors:** Kathrin Thedieck, Pazit Polak, Man Lyang Kim, Klaus D. Molle, Adiel Cohen, Paul Jenö, Cécile Arrieumerlou, Michael N. Hall

**Affiliations:** Biozentrum, University of Basel, Basel, Switzerland; National Institutes of Health, United States of America

## Abstract

TOR (Target of Rapamycin) is a highly conserved protein kinase and a central controller of cell growth. TOR is found in two functionally and structurally distinct multiprotein complexes termed TOR complex 1 (TORC1) and TOR complex 2 (TORC2). In the present study, we developed a two-dimensional liquid chromatography tandem mass spectrometry (2D LC-MS/MS) based proteomic strategy to identify new mammalian TOR (mTOR) binding proteins. We report the identification of Proline-rich Akt substrate (PRAS40) and the hypothetical protein Q6MZQ0/FLJ14213/CAE45978 as new mTOR binding proteins. PRAS40 binds mTORC1 via Raptor, and is an mTOR phosphorylation substrate. PRAS40 inhibits mTORC1 autophosphorylation and mTORC1 kinase activity toward eIF-4E binding protein (4E-BP) and PRAS40 itself. HeLa cells in which PRAS40 was knocked down were protected against induction of apoptosis by TNFα and cycloheximide. Rapamycin failed to mimic the pro-apoptotic effect of PRAS40, suggesting that PRAS40 mediates apoptosis independently of its inhibitory effect on mTORC1. Q6MZQ0 is structurally similar to proline rich protein 5 (PRR5) and was therefore named PRR5-Like (PRR5L). PRR5L binds specifically to mTORC2, via Rictor and/or SIN1. Unlike other mTORC2 members, PRR5L is not required for mTORC2 integrity or kinase activity, but dissociates from mTORC2 upon knock down of tuberous sclerosis complex 1 (TSC1) and TSC2. Hyperactivation of mTOR by TSC1/2 knock down enhanced apoptosis whereas PRR5L knock down reduced apoptosis. PRR5L knock down reduced apoptosis also in mTORC2 deficient cells. The above suggests that mTORC2-dissociated PRR5L may promote apoptosis when mTOR is hyperactive. Thus, PRAS40 and PRR5L are novel mTOR-associated proteins that control the balance between cell growth and cell death.

## Introduction

TOR (Target of Rapamycin) kinase is a highly conserved, central controller of cell growth [1]–[3]. The fundamental importance of TOR is underscored by genetic studies showing TOR to be essential for cell growth and development; disruption of the *TOR* gene is lethal in all examined species [4]–[12]. In humans, dysfunctional mTOR signaling plays an important role in many if not most cancers, as well as in diseases such as tuberous sclerosis complex (TSC, #191100 OMIM) and lymphangiomyelomatosis (LAM, #606690 OMIM). TOR is found, from yeast to human, in two functionally and structurally distinct multiprotein complexes termed TOR complex 1 (TORC1) and TORC2 [13]–[15]. The rapamycin-sensitive mammalian TOR complex 1 (mTORC1) consists of mTOR, mLST8 and Raptor [13], [16], [17]. mTORC2 contains Rictor and SIN1 instead of Raptor, and is rapamycin-insensitive [14], [15], [18]–[20]. Knock out of Raptor, SIN1 or Rictor in mice is embryonic lethal, indicating that both mTORC1 and mTORC2 are essential [19]–[22].

mTORC1 is activated by nutrients (amino acids), anabolic growth factors (e.g., insulin and insulin-like growth factor), and cellular energy (ATP) [1]–[3]. The growth factor signal and energy status are transmitted to mTORC1 via the essential tumor suppressor tuberous sclerosis complex (TSC) proteins TSC1 and TSC2 [23], [24]. The TSC heterodimer (TSC1-TSC2) is a GTPase activating protein (GAP) that inhibits the essential small GTPase Rheb [25], [26]. Rheb-GTP binds and activates mTORC1 [27]. Akt (also known as PKB) phosphorylates and inactivates TSC2 in response to growth factors [28], whereas AMPK phosphorylates and activates TSC2 in response to low energy (high AMP) [29], [30]. Nutrients impinge on mTORC1 at the level of Rheb or mTORC1 by a poorly understood mechanism involving the type III PI3K hVps34 [27], [31]. The upstream regulators of the more recently identified mTORC2 are not known, but mTORC2 appears to respond at least to growth factors, possibly via TSC1-TSC2 [32].

mTORC1 and mTORC2 separately control many cellular processes that collectively determine cell growth and development. mTORC1 controls transcription, protein synthesis, ribosome biogenesis, nutrient transport, and autophagy, among other processes. mTORC1 controls protein synthesis via phosphorylation of S6 kinase (S6K) and eIF-4E binding protein (4E-BP), two key regulators of translation initiation [3], [33], [34]. mTORC2 controls organization of the actin cytoskeleton via small Rho-type GTPases and Protein Kinase C [14], [15], [35], and thereby determines the shape and possibly motility of the cell. In addition, mTORC2 phosphorylates Ser473 in the hydrophobic motif of Akt and thereby activates Akt toward substrates such as the Forkhead transcription factor FOXO and the apoptosis regulator BAD [19]–[21], [36].

Although upstream regulators of mTOR, at least for mTORC1, are relatively well characterized, astonishingly few direct substrates and downstream effectors of the mTORCs are known. This is particularly true for mTORC2 which was discovered only recently and, due to its rapamycin insensitivity, is not pharmacologically addressable. To identify additional regulators, substrates, and/or components of the mTORCs, we developed a highly sensitive mass spectrometry-based screen. Here we report the identification of two novel mTOR binding proteins, PRAS40 (Q96B36 Swiss-Prot) and PRR5L (Q6MZQ0 Swiss-Prot), which bind specifically to mTORC1 and mTORC2, respectively. We further characterize the roles of these two proteins in mTOR complex formation and function.

## Results and Discussion

### PRAS40 and PRR5L bind specifically to mTORC1 and mTORC2

To identify new mTOR binding proteins, we used a ‘gel-less’ mass spectrometry-based method to screen for mTOR associated proteins. mTOR complexes, first purified by large scale immunoprecipitations (IPs) with antibody directed against mTOR, were digested with trypsin and after detergent removal subjected directly to two-dimensional liquid chromatography tandem mass spectrometry (2D LC-MS/MS). Within a single 2D LC-MS/MS run up to 270 different proteins were identified. To identify specific mTOR interactors, we compared mTOR and mock IPs, and chose those proteins that were present only in the mTOR IPs. Furthermore, to qualify as a specific interactor, a protein had to be identified in at least three out of four independent mTOR IP experiments. We reproducibly identified all known members of the mTOR complexes (Table 1). The sequence coverage for mTOR, Rictor, mLST8 and Raptor was about 20%, while sequence coverage for SIN1 was 13.8%. The above experiment was repeated with antibodies specific for Rictor or Raptor and, as expected, mTOR and mLST8 were found in both the Rictor and Raptor IPs, whereas SIN1 was found only in the Rictor IP.

**Table 1:** mTORC1 and mTORC2 associated proteins identified by 2D-LC-MS/MS

Protein	# Identifications (out of 4 independent IPs)	Sequence Coverage	Identified in IP of			Predicted MW (Da)	Length (aa)
			mTOR	Rictor	Raptor		
**mTOR**	4	26.2%	x	x	x	288892	2549
**Rictor**	4	21%	x	x		192217	1708
**Raptor**	4	18.2%	x		x	149038	1335
**SIN1**	4	13.8%	x	x		59123	522
**PRR5L**	3	5.2%	x	x		40866	368
**mLST8**	4	21.5%	x	x	x	35902	326
**PRAS40**	3	10.6%	x		x	27383	256

mTOR complexes were purified by immunoprecipitation (IP) with antibody directed against mTOR, Rictor or Raptor. Immunoprecipitates were analyzed by 2D LC-MS/MS. Proteins that were found in at least three out of four mTOR IPs, but not in mock IPs, were considered specific. IPs with antibodies directed against Rictor or Raptor indicated whether a candidate was specific for mTORC1 or mTORC2, respectively.

**Figure 1:**
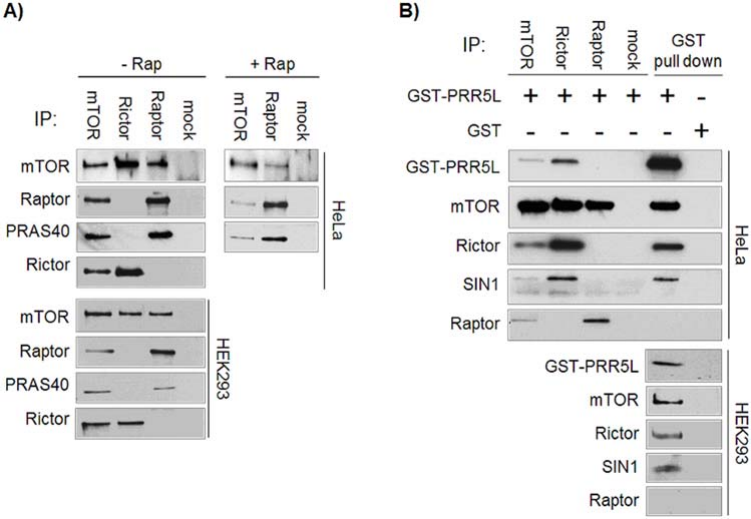
Confirmation of PRAS40 and PRR5L binding to mTOR. A. PRAS40 is associated specifically with mTORC1. mTOR, Rictor, Raptor and mock IPs were performed with HeLa and HEK293 extracts and analyzed by immunoblotting. PRAS40 was found specifically in mTOR and Raptor IPs. 1 h rapamycin treatment of cells dissociated a Raptor-PRAS40 subcomplex from mTOR. B. PRR5L is associated specifically with mTORC2. HeLa or HEK293 cells were transfected with GST-PRR5L or the empty plasmid. mTOR, Rictor, Raptor and mock IPs and GST pull downs were analyzed by immunoblotting. GST-PRR5L is detected specifically in mTOR and Rictor IPs. mTOR, Rictor and SIN1, but not Raptor, are detected specifically in GST-PRR5L pull downs.

In addition to the known mTORC partners, we also identified novel mTOR interacting proteins. The proline-rich Akt substrate PRAS40 (10.6% sequence coverage) was found in mTOR and Raptor IPs but not in Rictor IPs (Table 1). These interactions were confirmed by co-IP experiments with HeLa and HEK293 cells (Figure 1). PRAS40 is therefore a specific mTORC1 binding partner (Figure 1A). PRAS40 was originally discovered as an Akt substrate of unknown function [37]. During the preparation of this manuscript, two studies appeared suggesting that PRAS40 is an mTORC1 inhibitor [38], [39]. In addition to PRAS40, we identified the hypothetical protein Q6MZQ0/FLJ14213/CAE45978 as a specific mTORC2 interactor (Table 1). Q6MZQ0 was cloned with an N-terminal GST tag and its interaction with mTORC2 was confirmed by co-IP and GST pull downs from HeLa and HEK293 cells (Figure 1B). Since Q6MZQ0 displays 39% sequence similarity with the proline rich protein PRR5, we named it PRR5-Like protein (PRR5L). PRR5L is an uncharacterized protein. The related protein PRR5, however, is highly expressed in kidney and has been suggested to be a tumor suppressor since it is down regulated in a subset of breast tumors [40].

Earlier studies on the mTORCs failed to detect PRAS40 and PRR5L possibly because both have an apparent molecular weight of approximately 40 kDa as measured by SDS-PAGE. Former searches for mTOR binding proteins relied on IPs followed by SDS-PAGE analysis. The co-migrating heavy chain of the IP antibody used in these earlier experiments might have masked PRAS40 and PRR5L. Our 2D LC-MS/MS approach also identified Transferrin Receptor 1 (P02786 Swiss-Prot), NICE-4 (Q14157 Swiss-Prot), Plectin 1 (Q6S383 Swiss-Prot), and Thymopoietin (P42166 Swiss-Prot) as potential mTOR binding proteins, but direct co-IP experiments indicated that these were non-specific binding proteins (data not shown).

### PRAS40 binds mTORC1 via Raptor

**Figure 2:**
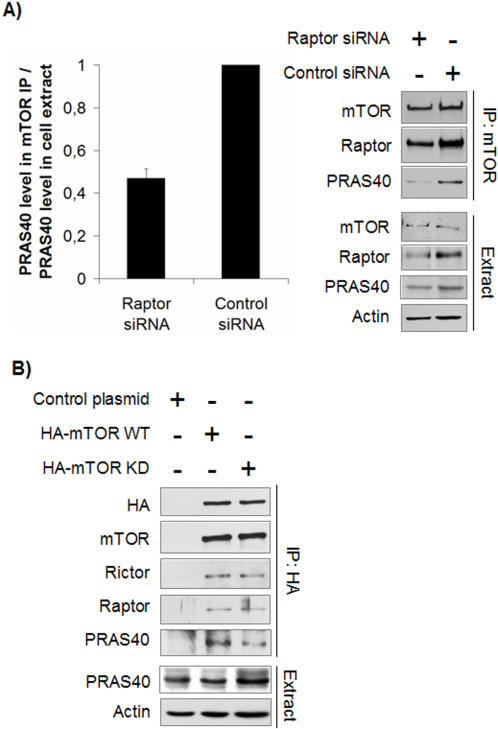
PRAS40 interacts with mTORC1. A. PRAS40 binds mTOR via Raptor. HEK293 cells were transfected with Raptor or control siRNA vectors and incubated for 4 days, followed by mTOR IP and immunoblotting. mTOR IPs and cell extracts were probed with antibodies directed against the indicated proteins. Since knock down of Raptor reduced the cellular amount of PRAS40, the PRAS40 signal in the IPs was quantified relative to PRAS40 levels in the corresponding extract. Quantitations were averaged over three independent experiments. Raptor knock down reduced the amount of PRAS40 associated with mTOR by 50%, as compared to control cells. B. mTOR kinase domain is involved in PRAS40 binding. HEK293 cells were transfected with a plasmid expressing wild type (WT) HA-mTOR or kinase dead (KD) HA-mTOR or an empty control plasmid, and incubated for 48 h followed by extract preparation and IP with an anti-HA antibody. PRAS40 levels in the extracts remained unaltered. PRAS40 association with mTORC1 containing HA-mTOR KD was moderately reduced as compared with mTORC1 containing HA-mTOR WT.

Following rapamycin treatment, PRAS40 dissociated from mTOR (Figure 1A). However, released PRAS40 remained bound to Raptor which, as reported previously [41], is also released from mTOR upon rapamycin treatment. Furthermore, PRAS40 binding to mTOR was strongly reduced when Raptor was knocked down (Figure 2A). These findings indicate that PRAS40 binds mTORC1 via Raptor. We also observed that PRAS40 associated less well with a kinase dead version of mTOR (Figure 2B), suggesting that mTORC1-mediated phosphorylation of PRAS40 (see below) may affect the PRAS40- mTORC1 interaction. Our findings are in agreement with recent PRAS40 studies showing that PRAS40 binds preferentially to Raptor [38], and that the mTOR kinase domain is also involved in PRAS40 binding [39].

### PRAS40 is a substrate and an inhibitor of mTORC1 *in vitro*

**Figure 3:**
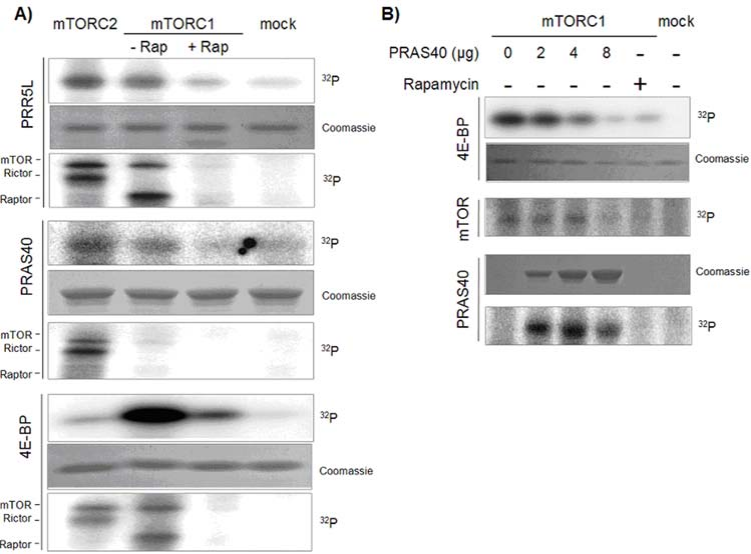
mTOR phosphorylates PRAS40 and PRR5L, and PRAS40 inhibits mTOR kinase activity. A. mTOR phosphorylates PRAS40 and PRR5L. Kinase assays were performed using mTORC1 or mTORC2 immunopurified from HEK293 cells, and purified PRAS40, GST-PRR5L (PRR5L) or 4E-BP as substrates. Rapamycin (100 nM) and purified FKBP12 were added directly to the reaction. Both PRR5L and PRAS40 are phosphorylated *in vitro* by both mTORCs. Phosphorylation by mTORC1 was rapamycin-sensitive. B. PRAS40 inhibits mTORC1 kinase activity toward 4E-BP and PRAS40 itself. Kinase assays were performed using mTORC1 immunopurified from HEK293 cells, purified 4E-BP as a substrate, and increasing concentrations of PRAS40. PRAS40 inhibits mTORC1 autophosphorylation and mTORC1 phosphorylation of 4E-BP and PRAS40, in a concentration-dependent manner.

The above results suggested that PRAS40 is phosphorylated by mTORC1. To investigate if PRAS40 is an mTORC1 substrate, we performed *in vitro* kinase assays with mTORC1 or mTORC2 and purified PRAS40. PRAS40 was phosphorylated weakly by both mTORC1 and mTORC2. Importantly, we also found that PRAS40 inhibited mTORC1 autophosphorylation but not mTORC2 autophosphorylation (Figure 3A), suggesting that the weak phosphorylation of PRAS40 by mTORC1 might be due to PRAS40-mediated inhibition of mTORC1. To investigate whether PRAS40 inhibits mTORC1 kinase activity, we performed *in vitro* kinase assays with the known mTORC1 substrate 4E-BP and increasing concentrations of purified PRAS40. PRAS40 inhibited both mTORC1 autophosphorylation and mTORC1 phosphorylation of 4E-BP, in a concentration dependent manner (Figure 3B). In addition, PRAS40 phosphorylation inversely correlated with the concentration of PRAS40 in the kinase reaction, suggesting that PRAS40 is indeed both a substrate and an inhibitor of mTORC1 kinase activity. Our finding that PRAS40 inhibits mTORC1 kinase activity toward 4E-BP and PRAS40 is in agreement with the observation of Sancak et al. [38] and Vander Haar et al. [39] that PRAS40 inhibits mTORC1 toward S6K1. Hence, we conclude that PRAS40 is a broad mTORC1 inhibitor that inhibits mTORC1 kinase activity toward itself, 4E-BP, S6K1, and PRAS40. It remains to be determined whether mTORC1-mediated phosphorylation of PRAS40 plays a role in PRAS40's ability to bind and inhibit mTORC1.

Interestingly, we found that phosphorylation of the Akt consensus site T246 in PRAS40 is moderately reduced in Rictor knock down cells (data not shown). This suggests that mTORC2 may activate Akt toward PRAS40. This in turn suggests that mTORC2, via PRAS40, may be upstream of mTORC1. If mTORC2 is indeed upstream of mTORC1, it might be only under specific conditions or only with regard to particular mTORC1 substrates (other than S6K1), as we and others failed to detect an effect of mTORC2 disruption on S6K1 phosphorylation [14], [15]. The potential regulation of mTORC1 by mTORC2 requires further investigation.

### PRAS40 deficiency prevents induction of apoptosis by TNFα and cycloheximide

**Figure 4:**
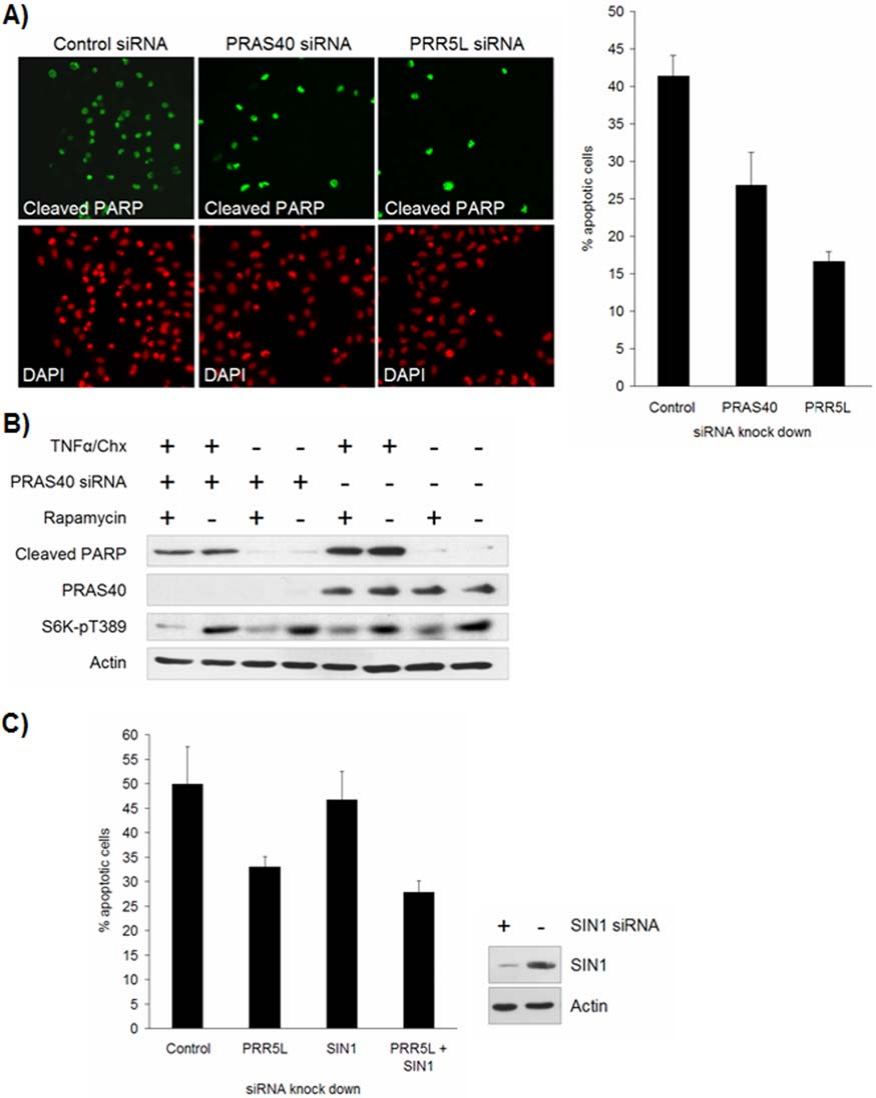
PRAS40 and PRR5L are pro-apoptotic. A. PRR5L and PRAS40 knock down cells are resistant to TNFα/cycloheximide induced apoptosis. HeLa cells were transfected with PRR5L, PRAS40 or control siRNA and incubated for 48 h, followed by 2 h induction of apoptosis with TNFα and cycloheximide. Cells were fixed and stained with DAPI and cleaved PARP antibody, and the percentage of apoptotic cells was quantified. B. PRAS40's effect on apoptosis is independent of mTORC1. HeLa cells were transfected with PRAS40 or control siRNA and incubated for 48 h, and treated with 100 nM rapamycin or carrier for 1 h before incubation with TNFα and cycloheximide for 2 h to induce apoptosis. Extracts were analyzed by immunoblotting with the indicated antibodies. C. PRR5L deficiency protects against apoptosis in SIN1 deficient cells. HeLa cells were transfected with diced PRR5L siRNA and/or synthetic siRNA against SIN1 as indicated, or the appropriate control siRNAs. Cells were incubated for 48 h, and apoptosis was induced with TNFα and cycloheximide for 2 h. Cells were fixed and stained with DAPI and cleaved PARP antibody, and the percentage of apoptotic cells was quantified. The efficiency of SIN1 knock down was assessed in parallel by immunoblotting (right panel).

Constitutively active mTOR reduces apoptosis [42] whereas inhibition of mTORC1 with rapamycin induces or facilitates apoptosis in several cell lines [43]–[48]. We therefore reasoned that the mTORC1 inhibitor PRAS40 might promote apoptosis and that PRAS40 knock down would thus protect cells against the induction of apoptosis. To investigate this possibility, we examined the effect of PRAS40 knock down on the sensitivity of HeLa cells to apoptosis induction by TNFα in combination with cycloheximide. To monitor apoptosis, treated cells were processed for visualization of DNA and cleaved PARP. We found that apoptosis was reduced in PRAS40 knock down cells (Figure 4A), suggesting that PRAS40 is indeed pro-apoptotic. To analyze if PRAS40 promotes apoptosis via its inhibitory effect on mTORC1, we investigated if rapamycin suppressed the effect of a PRAS40 deficiency on TNFα/cycloheximide induced apoptosis in HeLa cells. Rapamycin failed to prevent the reduction in apoptosis caused by PRAS40 knock down (Figure 4B). In addition, rapamycin treatment did not affect apoptosis induction by TNFα/cycloheximide in control cells (Figure 4B), even after 6h of rapamycin treatment (data not shown). The finding that rapamycin failed to mimic the pro-apoptotic effect of PRAS40, suggests that PRAS40 mediates apoptosis independently of its inhibitory effect on mTORC1. However, PRAS40 T246 phosphorylation (PRAS40-pT246) appears to protect neuronal cells from apoptosis after stroke [49]. PRAS40-pT246 has also been proposed to promote cell survival in cancer cells [50]. The fact that T246 is the site via which Akt negatively regulates PRAS40's ability to inhibit mTORC1 [38] suggests that PRAS40 may indeed be pro-apoptotic via its ability to inhibit mTORC1. To explain the apparent discrepancy with our inability to induce apoptosis with rapamycin, PRAS40 may have to inhibit both mTORC1 and a second, unknown rapamycin insensitive target (or only this second target) to perform its pro-apoptotic function. mTORC1 may be the more important target in those cell lines where rapamycin induces or facilitates apoptosis [43]–[48].

### PRR5L binds mTORC2 via Rictor/SIN1

**Figure 5:**
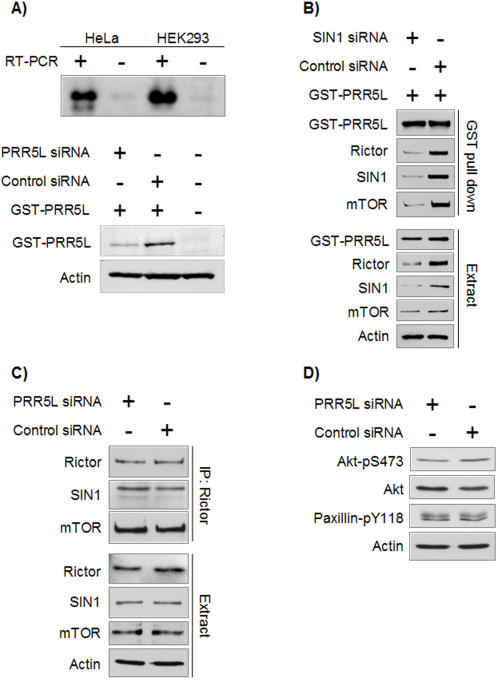
PRR5L binds to mTORC2 via SIN1 and/or Rictor but does not affect mTORC2 integrity or kinase activity. A. PRR5L expression in HeLa and HEK293 cells, and PRR5L knock down efficiency. Top panel: Total RNA was purified from HeLa or HEK293 cells, followed by reverse transcription and PCR with primers corresponding to PRR5L. As a negative control, reverse transcription without the transcriptase enzyme was performed. Endogenous PRR5L is expressed in both cell lines. Bottom panel: HEK293 cells were cotransfected with a GST-PRR5L vector and PRR5L siRNA or control siRNA, and incubated for 48 h. Immunoblots were performed on with antibody against GST or Actin. B. PRR5L binds mTOR via SIN1 and/or Rictor. HEK293 cells were cotransfected with a GST-PRR5L vector and a SIN1 siRNA vector or a control siRNA vectors, and incubated for 4 days. GST pull downs were immunoblotted with the indicated antibodies. GST-PRR5L was detected with an anti-GST antibody. mTOR binding to GST-PRR5L is weaker in the absence of SIN1 and Rictor. C. mTORC2 remains intact in PRR5L knock down cells. HEK293 cells were transfected with PRR5L siRNA or control siRNA and incubated for 48 h. Rictor IPs were immunoblotted with the indicated antibodies. D. mTORC2 readouts are unaltered in PRR5L knock down cells. HEK293 cells were transfected with PRR5L siRNA or control siRNA and incubated for 48 h. Immunoblots were performed on protein extracts with the indicated antibodies. The phosphorylation of Akt S473 and paxillin Y118 is unaltered by PRR5L knock down.

Since we initially identified endogenous PRR5L as an mTORC2 binding protein in HeLa cells, we verified PRR5L expression in HEK293 cells by RT-PCR (Figure 5A). PRR5L is strongly expressed in both HeLa and HEK293 cells. Subsequent experiments were performed with HEK293 cells due to the higher transfection efficiency with these cells. To investigate whether PRR5L binds directly to mTOR or via other mTORC2 members, we examined PRR5L binding to mTOR in SIN1 knock down cells. As reported previously [18], [19], we observed a reduction in the amount of Rictor when SIN1 was knocked down (Figure 5B), supporting the earlier suggestion that these two proteins stabilize each other. We found that the amount of mTOR in GST-PRR5L pull downs from the SIN1/Rictor deficient cells was substantially reduced compared to control cells (Figure 5B). This suggests that PRR5L binds to mTOR via Rictor and/or SIN1. To investigate whether PRR5L is required for mTORC2 integrity, we examined the binding of mTOR and SIN1 to Rictor in cells knocked down for PRR5L (Figure 5C). The amount of mTOR and SIN1 bound to Rictor, as measured by co-IP, remained unchanged in PRR5L knock down cells. We then investigated if PRR5L is required for mTORC2 kinase activity. Phosphorylation of Akt S473 and Paxillin Y118 is reduced upon mTORC2 disruption [14], [15]. However, in PRR5L deficient cells the levels of Akt-pS473 and Paxillin-pY118 remained unchanged (Figure 5D). The findings that PRRL5 is not required for mTORC2 integrity or for phosphorylation of known mTORC2 targets suggest that PRR5L is not an mTORC2 upstream regulator or an integral component of mTORC2.

### PRR5L is phosphorylated by mTOR *in vitro*

To determine whether PRR5L is a phosphorylation substrate for mTOR, we performed *in vitro* kinase assays with mTORC1 or mTORC2 and purified PRR5L. We found that PRR5L is phosphorylated by both mTORC1 and mTORC2, the former but not the latter phosphorylation being sensitive to rapamycin treatment (Figure 3A). Hence, PRR5L might be regulated through mTOR phosphorylation. PRR5L addition did not affect mTORC1 or mTORC2 autophosphorylation (Figure 3A), suggesting that PRR5L does not function as an mTORC2 inhibitor like PRAS40 for mTORC1. The significance of the phosphorylation of PRR5L by mTOR, in particular by mTORC1, remains to be determined. The above results taken together suggest that PRR5L is a downstream effector of mTORC2.

### PRR5L dissociates from mTORC2 in TSC1/2 deficient cells

**Figure 6:**
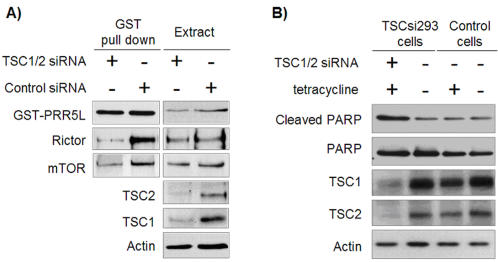
Analysis of TSC1/2 knock down cells. A. PRR5L is released from mTORC2 in TSC1/2 deficient cells. TSC knock down was induced in TSCsi293 cells by tetracycline treatment for 4 days. Cells were cotransfected with GST-PRR5L vector and incubated for 2 days, followed by GST pull downs and immunoblots with the indicated antibodies. GST-PRR5L was detected with an anti-GST antibody. B. TSC1/2 knock down facilitates apoptosis. TSCsi293 and T-REx-293 (control) cells were treated with tetracycline for 4 days. Apoptosis was induced by 1.5 h treatment with TNFα and cycloheximide. Extracts were probed with the indicated antibodies.

To investigate whether TSC1-TSC2 influences PRR5L binding to mTORC2, we examined GST-PRR5L pull downs from TSC1/2 deficient cells. TSC1/2 deficient cells exhibited reduced amounts of Rictor and mTOR bound to GST-PRR5L (Figure 6A). The above finding suggests that PRR5L dissociates from mTORC2 in cells with hyperactive mTOR signaling. It remains to be determined whether the effect of TSC1/2 knock down on PRR5L binding is via mTORC1 or mTORC2. TSC1/2 knock down hyperactivates mTORC1 [1]–[3] and possibly also mTORC2 (K.D.M and M.N.H., unpublished).

### PRR5L promotes apoptosis

Hyperactive mTOR signaling, in TSC knock out MEFs, enhances induction of apoptosis by FCS starvation or TNFα/cycloheximide [51], [52]. In agreement, we found that TSC1/2 knock down in human cells, in which PRR5L dissociates from mTORC2, also enhances TNFα/cycloheximide induced apoptosis (Figure 6B). To investigate whether PRR5L plays a role in apoptosis, we examined if PRR5L knock down affects TNFα/cycloheximide induced apoptosis. PRR5L knock down cells were less apoptotic compared to control cells, at various time points after TNFα/cycloheximide treatment, suggesting that PRR5L is pro-apoptotic (Figure 4A and S1). The finding that PRR5L is pro-apoptotic is consistent with the suggested role of the related protein PRR5 as tumor suppressor [40]. Furthermore, the observation that PRR5L is pro-apoptotic and is released from mTORC2 in cells with enhanced apoptosis (TSC1/2 deficient cells) suggests that released PRR5L may promote apoptosis. This in turn suggests that PRR5L is downstream of mTORC2 in mediating apoptosis. To test this possibility, we investigated whether a PRR5L deficiency still reduces apoptosis in cells knocked down for TORC2. We found that cells knocked down for both PRR5L and the mTORC2 component SIN1 were similar to cells knocked down only for PRR5L, with regard to induction of apoptosis by TNFα/cycloheximide (Figure 4C). This observation is consistent with a model in which PRR5L acts downstream of mTORC2. In particular, in response to hyperactive mTOR signaling, PRR5L may dissociate from mTORC2 to promote apoptosis. However, our data do not rule out the possibility that PRR5L controls apoptosis independently of mTORC2. It is important to note that mTORC2 also promotes cell survival via a mechanism other than tethering PRR5L. mTORC2 phosphorylates and activates Akt which then phosphorylates and inactivates the pro-apoptotic factors BAD and FOXO1/3a [19], [20], [36]. The above taken together suggests that either too much or too little mTOR signaling predisposes a cell to apoptosis. There seems to be a delicate balance between cell growth and cell death that may be mediated at least in part by PRR5L.

In summary, we describe two new mTOR interactors, PRAS40 and PRR5L. PRAS40 binds specifically to mTORC1 whereas PRR5L is mTORC2 specific. PRAS40 binding to mTORC1 is primarily via Raptor but also requires mTOR kinase activity. mTORC1 phosphorylates PRAS40 and this phosphorylation may contribute to the mTORC1-PRAS40 interaction. Furthermore, PRAS40 inhibits mTORC1 autophosphorylation and mTORC1 kinase activity toward its substrates 4E-BP and PRAS40. This observation extends two recent studies showing that PRAS40 inhibits mTORC1 toward its substrate S6K [38], [39]. Thus, PRAS40 is an upstream negative regulator of mTORC1. We also show that PRAS40 is pro-apoptotic, but this may be an mTORC1 independent function of PRAS40. PRR5L, the new mTORC2-specific interactor, binds mTOR via SIN1 and/or Rictor. Unlike Rictor and SIN1, PRR5L is not required for mTORC2 integrity or mTORC2 kinase activity toward its downstream readouts Akt and Paxillin. Furthermore, we observed that PRR5L binding to mTORC2 is reduced in TSC1/2 deficient cells. We conclude that PRR5L dissociates from mTORC2 in cells with hyperactive mTOR signaling. We show that a TSC1/2 deficiency enhances TNFα/cycloheximide induced apoptosis. Conversely, knock down of PRR5L prevents apoptosis, even in mTORC2 deficient cells. We suggest that PRR5L is downstream of mTORC2 and is pro-apoptotic. It will be of interest to determine whether PRR5L is a tumor suppressor as suggested for the related protein PRR5 [40].

## Materials and Methods

### Screen for mTOR binding proteins and mass spectrometry

mTOR binding proteins were purified essentially as reported [14]. For each IP experiment, 6 10 cm dishes of HeLa cells at 70% confluence were used. IPs were performed with 6 µg of mTOR (Santa Cruz), Rictor or Raptor (Bethyl), or control goat (Santa Cruz) or rabbit (Bethyl) antibodies. Antibodies were bound to 300 µL magnetic Protein G coupled Dynabeads (Invitrogen). Digestion was performed on the beads [53] with 1 µg of Trypsin (Promega). After drying, detergents were removed by hydrophilic interaction chromatography on PolyHYDROXYETHYL TopTips (PolyLC Inc.) according to the manufacturer's instructions. Ammonium acetate remainders were removed by repeated drying of the samples.

### LC-MS/MS

The peptides were analyzed by two-dimensional capillary liquid chromatography and tandem MS using a PolySULFOETHYL A ion-exchange column (0.15×50 mm, PolyLC, Columbia, MD), connected in series to a C18 trap column (Zorbax 300SB, 0.3×50 mm, Agilent Technologies, Basel, Switzerland), and to a Magic C18 separation column (0.1×100 mm, Thermo Scientific, Basel, Switzerland). The peptides were injected first onto the cation exchange column. Unadsorbed peptides were trapped on the Zorbax column and eluted onto the separation column with a linear 75 min gradient from 2 to 75% B (0.1% acetic acid in 80% acetonitrile) in solvent A (0.1% acetic acid in 2% acetonitrile). Next, peptides that had been retained by the ion-exchange column were sequentially eluted and trapped on the C18 trap column with 10 mL pulses of 50, 100, 150, 200, 250, 300, 350, 400, and 500 mM ammonium acetate, pH 3.3. Peptides eluted by each individual salt pulse were separated by the acetonitrile gradient as described above. The flow was delivered with a Rheos 2200 HPLC system (Thermo Scientific, Basel, Switzerland) at 50 mL/min. A precolumn splitter reduced the flow to approximately 500 nl/min. The eluting peptides were ionized by a Finnigan nanospray ionization source (Thermo Scientific, Basel, Switzerland). The LTQ orbitrap instrument was operated in the data-dependent mode. A survey scan was performed in the Orbitrap between *m/z* 400-1600 Da at 60,000 resolution. The three most abundant ions detected were fragmented in the LTQ mass spectrometer and mass analyzed in the Orbitrap at a resolution of 7,500. Singly charged ions were not subjected to fragmentation. The normalized collision energy was set to 35%. Individual MS/MS spectra were searched against the NCBI non-redundant databank using the TurboSequest software [54]. The Sequest filter parameters were as follows: Xcorr versus charge state was 1.50 for singly, 2.00 for doubly, and 2.50 for triply charged ions, respectively; the ΔCN was 0.1, and the protein probability was set to 0.01.

### Plasmids and reagents

Rictor, Raptor and SIN1 siRNA constructs were previously described [14], [20]. An empty pSuper-GFP-neo construct was used as a control. HA-mTOR and kinase dead HA-mTOR constructs were a kind gift from Dr. G. Thomas and were described previously [29], the control empty vector was created by cutting out the mTOR fragment using NotI and PstI.

The coding region of PRR5L was cloned from human cDNA made from HeLa cells, using the following primers: 5′ ATG ACC CGC GGC TTC G 3′ (forward, contained also restriction sites for either BamHI or SpeI), and 5′ T CAG CTG AGG GAA GCA CAG 3′ (reverse, contained also a NotI restriction site). The PCR product was digested either with BamHI and NotI or with SpeI and NotI, and cloned into pGEX-6P-1 or pEBG, respectively. Recombinant GST-PRR5L was expressed from pGEX-6P-1 and purified from *E. coli.*

The generation of the inducible TSC knock down cell line TSCsi293 from an HEK293 cell line (T-REx-293, Invitrogen), that expresses the tetracycline repressor protein TetR, and its handling were as described (K. D. M. and M. N. H., submitted). Cycloheximide was dissolved in water and used at a final concentration of 2.5 µg/mL (Calbiochem), rapamycin was dissolved in DMSO and used at a final concentration of 100 nM (LC laboratories), TNFα was dissolved in PBS containing 0.1% BSA, and used at a final concentration of 10 ng/mL (R&D systems). Purified PRAS40 was from Biosource, purified 4E-BP was from Stratagene.

### RNA interference

siRNAs against PRAS40 and PRR5L and control siRNA against Luciferase were generated as described [55] using the following reagents: 5× Megascript T7 Kit (Ambion); Turbo Dicer siRNA Generation Kit (Genlantis); RNA Purification System (Invitrogen). Primers for PRAS40 were as follows: gene specific primers: forward: ttgcctccacgacatcgcac, reverse: tatttccgcttcagcttctgg, T7 primers: forward: gcgtaatacgactcactataggccacagggctgccactg, reverse: gcgtaatacgactcactataggaagtcgctggtgttaagcc. Primers for PRR5L were as follows: gene specific primers: forward: tcgtccattgtccagatgttg, reverse: agctgagggaagcacagttc, T7 primers: forward: gcgtaatacgactcactataggctcatcctgcagagtgttc, reverse: gcgtaatacgactcactataggagctccgagccctcctg. For SIN1 knock down, a synthetic pool siRNA or the appropriate control pool siRNA (Dharmacon) were used as described [19].

### Cell culture and transfections

HEK293 and HeLa cells were maintained in DMEM containing 10% fetal bovine serum. Small RNAi was transfected with INTERFERin (Polyplus transfection). For combined transfection of small RNAi and DNA, jetSI-ENDO (Polyplus transfection) was used. DNA was transfected using either lipofectamin (Invitrogen) or jetPEI (Polyplus transfection). All transfections were done according to the manufacturers' instructions, for 48 h for expression or small RNAi, or for 4 days in the case of pSuper-based siRNA. Cells were harvested with lysis buffer that contained 40mM HEPES pH 7.5, 120 mM NaCl, 1 mM EDTA, 0.3% CHAPS, supplemented with protease inhibitor cocktail (Roche). In the cases where phosphorylation was to be detected, the lysis buffer was also supplemented with10 mM NaF, 10 mM NaN3, 10 mM p-nitrophenylphosphate, 10 mM sodium pyrophosphate, and 10 mM beta-glycerophosphate. Lysates were incubated for 20 minutes on ice, then cleared by a spin at 600 g for 3 minutes. Supernatants were collected and used for immunoprecipitations, GST pull downs or immunoblots.

### RT-PCR

RNA was purified using the RNAeasy mini kit (Qiagen), according to the manufacturer's instructions. One microgram of total RNA was reverse transcribed using Superscript II reverse transcriptase (Invitrogen) and random nonamers (Sigma). The reverse transcription reaction was used as a template for PCR, with the PRR5L primers described above.

### Immunoblotting

Protein extracts were prepared as described [14], resolved on SDS-PAGE and transferred to PVDF membranes (Immobilon-P, Millipore). Immunoblots were performed using the following antibodies: Rictor, Raptor (Bethyl); mTOR (Santa Cruz); S6K, phospho-S6K, Akt, phospho-Akt (Thr308), phospho-Akt (Ser473), PARP, cleaved PARP (Cell signaling); PRAS40 (Biosource; PRAS40 antibodies were previously described [37]); SIN1 (kind gift from Dr. Bing Su, University of Yale, CT). GST-PRR5L was detected with a GST antibody (GE Healthcare), since no antibody for PRR5L was available.

### Apoptosis assay

RNAi experiments were performed by transfecting HeLa cells for 48 hours in a 96-well format. 48 hours after siRNA transfection, apoptosis was induced for the indicated times as described [56]. Apoptosis, in response to TNFα and cycloheximide treatment, was quantified by a cleaved PARP (cPARP) immunofluorescence assay (Cell Signaling). The assay was performed according to the manufacturer's instructions. Briefly, after apoptosis induction, cells were fixed with pre-chilled 100% methanol for 5min and then washed with 0.1% Triton X-100 and PBS sequentially. Cells were then incubated with anti-cPARP antibody (1/200 dilution) overnight at 4°C, washed and incubated for 1 hour with a mixed solution containing Alexa 568 goat anti-Rabbit antibody (1/500 dilution) and Hoechst (Invitrogen, 1/1000 dilution). Images were automatically taken by an ImageXpress Micro (Molecular Devices, Sunnyvale, USA). Apoptosis was quantified by automated image processing. The multi-wavelength cell scoring application module of the analysis software MetaXpress was used to quantify, at the single cell level, the intensity of cPARP staining (200 was used as the intensity above background for cPARP images). More than 6000 cells per condition were analyzed. For analysis by immunoblotting cPARP levels were detected by human or mouse specific antibodies (Cell Signaling). mTORC1 was inhibited by preincubation with 100 nM rapamycin for 1 h, and apoptosis was subsequently induced by TNFα/cycloheximide for 1.5 h in the presence of rapamycin.

### Immunoprecipitation and GST pull down

Immunoprecipitations were performed as described [14]. Pull downs of GST-PRR5L were similar to immunoprecipitations, with glutathione-coupled beads (GE Healthcare).

### Kinase assay

Kinase assays were performed as described [14].

## Supporting Information

Figure S1 Time course for apoptosis induction. HeLa cells were transfected with PRR5L or control siRNA and incubated for 48 h, followed by apoptosis induction for the indicated time spans by TNFalpha and cycloheximide. Cells were fixed and stained with cleaved PARP antibody, and the percent of apoptotic cells was quantified.
(0.64 MB TIF)
