# Supplementary figures and images for: PRAS40 and PRR5-Like Protein Are New mTOR Interactors that Regulate Apoptosis
Source: PLoS One. 2007 Nov 21;2(11):e1217. doi: 10.1371/journal.pone.0001217 (PMC2075366; doi:10.1371/journal.pone.0001217)

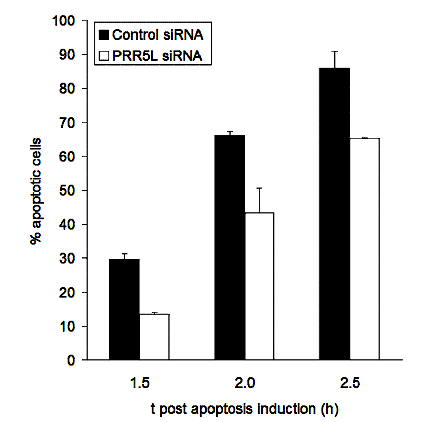

Supplement: Figure S1 — Time course for apoptosis induction. HeLa cells were transfected with PRR5L or control siRNA and incubated for 48 h, followed by apoptosis induction for the indicated time spans by TNFalpha and cycloheximide. Cells were fixed and stained with cleaved PARP antibody, and the percent of apoptotic cells was quantified. (0.64 MB TIF) [file pone.0001217.s001.tif]
